# Developing Physiotherapy in Primary Health Care: A First Snapshot from the Italian Metropolitan City of Milan

**DOI:** 10.3390/healthcare12161628

**Published:** 2024-08-15

**Authors:** Claudio Cordani, Sergio Perillo, Davide Corbetta, Elisabetta Sarasso, Federica Agosta, Massimo Filippi, Angelo G. Mazzali, Federico Pennestrì

**Affiliations:** 1 Department of Biomedical Surgical and Dental Sciences, University “La Statale”, 20122 Milan, Italy; 2 IRCCS Istituto Ortopedico Galeazzi, 20157 Milan, Italy; 3 Ordine Interprovinciale Della Professione Sanitaria di Fisioterapista di “Milano, Como, Cremona, Lecco, Lodi, Monza Brianza, Sondrio, Varese”, 26100 Cremona, Italy; 4 Department of Rehabilitation and Functional Recovery, IRCCS San Raffaele Scientific Institute, 20132 Milan, Italy; 5 Neuroimaging Research Unit, Division of Neuroscience, IRCCS San Raffaele Scientific Institute, 20132 Milan, Italy; 6 Department of Neuroscience, Rehabilitation, Ophthalmology, Genetics and Maternal Child Health, University of Genoa, 16132 Genoa, Italy; 7 Faculty of Medicine, Vita-Salute San Raffaele University, 20132 Milan, Italy; 8 Neurology Unit, IRCCS San Raffaele Scientific Institute, 20132 Milan, Italy; 9 Neurorehabilitation Unit and Neurophysiology Service, IRCCS San Raffaele Scientific Institute, 20132 Milan, Italy

**Keywords:** rehabilitation, primary health care, survey and questionnaires, health services, health policy

## Abstract

Introduction: Since the COVID-19 pandemic, the Italian National Health Service (NHS) has been undergoing a structural reform shifting focus from hospital-centered care to smaller, intermediate, or primary health facilities closer to the community (e.g., community hospitals and community houses). This reorganization should include rehabilitation and physiotherapy, but the actual spread of these services is still unclear. Objective: This study explored the number and characteristics of community-based physiotherapy services in the Metropolitan City of Milan (Italy). Methods: Between April and May 2024, we distributed a structured, anonymous online survey about community physiotherapy services and users to all Directors of the Health and Social Care Professions Departments (DAPSS) in the Metropolitan City of Milan. We used descriptive statistics to analyze the number of community houses offering physiotherapy services, the specific intervention areas, and access modalities. Results: Six out of seven DAPSS Directors completed the survey (87%). Thirty-seven community houses were reported in the area, with fourteen of these offering physiotherapy services. In most of them, physiotherapy was a primary reason for access following a general practitioner’s prescription. Five out of six responders reported that rehabilitation needs were mainly assessed by specialists in Physical and Rehabilitation Medicine, with physiotherapists involved in the assessment process in two cases. Physiotherapists primarily handled the intervention phase, dealing mainly with orthopedic and neurological conditions. DAPSS Directors noted that additional physiotherapy initiatives focusing on prevention will be implemented. Conclusions: Physiotherapy services are becoming available in the Metropolitan City of Milan. However, more efforts are needed to facilitate access and ensure tailored assessment and effective interventions, particularly in preventive care. Future investigations should help to better define the number and the characteristics of the patients who can most benefit from this type of care, the number of sessions they need, and with what types of intervention; it would be also necessary to better define the communication network in the area that allows doctors, health professionals, and patients to be informed about this possibility.

## 1. Introduction

In 2021, after the approval by the European Commission of the €807 billion temporary recovery instrument “Next Generation EU”, the Italian Government started its National Recovery and Resilience Plan (Piano Nazionale di Ripresa e Resilienza—PNRR) dedicating about €190 billion of the European Union (EU) funds to six strategic areas: digital innovation, ecological transition, infrastructures for mobility, education and research, social cohesion, and healthcare. Fifteen billion euros were allocated to this latter sector, paying particular attention to primary care enhancement (€7 billion) and the digitalization of the National Health Service (NHS) (about €8 billion) [1,2,3].

As agreed with the EU Council, the first healthcare milestone of the PNRR that had to be reached was the adoption of national legislation aimed at the “definition of a new organisational model for Territorial healthcare assistance network”, defining a regulatory framework that identifies structural, technological, and organizational standard, as well as a new institutional structure of Health-Environment-Climate prevention [4]. This requirement was matched by adopting the Minister of Health Decree n. 77 (23 May 2022), which reported the descriptions of the new territorial healthcare implementation program [3,5].

In particular, the Decree identified a new facility for primary care, the so-called community house. “The community house is the physical and easily identified place to which citizens can access for health, and social care needs and is the organizational model of outreach care for the target population. In the community house, all professionals work in an integrated and multidisciplinary manner for the design and delivery of health and social integration interventions” [5]. These facilities are further divided into hubs and spokes. Each hub community house has to cover 40–50,000 inhabitants and must provide basic diagnostic services aimed at chronicity monitoring, the continuity of care interventions, and biological sampling. Spoke facilities have fewer mandatory requirements delivering basic home care services, outpatient visits for high-prevalence diseases, visits booking, and nursing services [5].

In this context, rehabilitation professionals (including physiotherapists) can play a valuable role in delivering evidence-based intervention in terms of all of prevention, patient engagement, and functional recovery. For example, physiotherapists could deliver interventions to prevent low back pain recurrences, falls and subsequent traumatic injuries, and cardiovascular, as well as respiratory maintenance programs, providing positive clinical results and reducing the utilization of more complex and expensive healthcare services [6,7,8,9,10]. Unfortunately, the Decree does not define, as mandatory, the presence of physiotherapists inside these facilities, leaving the decision to the subjective initiatives of every single local health agency. 

Since the beginning of the PNRR, the number of community houses has continuously grown to reach the EU milestone [11]. However, the clinical characteristics of these new facilities are not precisely mapped. Specifically, no definite data are available about physiotherapy services in this setting, and the description of the real-world experience of a relevant Italian area could provide useful information to improve interventions not only in other parts of the country but also at the international level to stimulate the informed organization of new community-based initiatives.

In this research, we aimed at exploring the number and main characteristics of community house physiotherapy services in the Metropolitan City of Milan, which is a representative case study for the high number of patients it should cover.

## 2. Materials and Methods

### 2.1. Study Design

We conducted a cross-sectional study involving a structured online survey that was distributed through the “Interprovincial Order of Physiotherapists of Milan, Como, Cremona, Lecco, Lodi, Monza Brianza, Sondrio, Varese” (OFI). The Orders of Physiotherapists are the official institutional bodies (38 operating across the country at regional or provincial level) that legally recognize professional physical therapists allowed to practice in Italy. No incentives were offered to participants. We followed the Guidelines for Reporting Survey-Based Research [12,13] and Observational Studies [14] as reported in Supplementry Materials S1. The protocol was registered on Open Science Framework at the following link: https://osf.io/eswmr/ (accessed on 15 August 2024). No protocol amendments were made.

### 2.2. Selection Criteria

To be included the participants had to (i) be a Member of the Corporate Directorate of Health and Social Care Professions (DAPSS) in one of the seven Territorial Social-Health Authorities (ASST; the responsible bodies for the provision of hospital and primary care in the Lombardy Region) of the Metropolitan City of Milan and (ii) read and provide consent to the use of anonymous data in aggregated form for research purposes. 

### 2.3. Survey Management

We developed an original web-based questionnaire using Google Forms to collect data. We launched the survey from 1 April 2024 until 31 May 2024 through public institutional emails. Data were collected anonymously, and participants gave their permission for the aggregated publication.

### 2.4. Survey Questionnaire

We conducted a pilot test of our survey involving all the Governing Board members of the OFI, to assess its clarity and accuracy. After revision, the final version of the questionnaire consisted of 21 close-ended items regarding personnel and services of the community houses, as well as the kind of patients and the access modalities. All items were mandatory and were not grouped in specific sub-sections. To ensure that the questionnaire was well suited for collecting data from the target population, the questionnaire was developed in Italian (Supplementary Materials S2). An English-translated version is reported in Table 1 for display purposes.

### 2.5. Statistical Analysis

Descriptive statistics are presented as medians and interquartile ranges or absolute values, percentages, and frequencies, based on the nature of each variable. We reported the data in a narrative description or quantitative summary (table). We also looked into and registered the response rate. Data were analyzed using SPSS version 26.0 (IBM, Armonk, NY, USA).

## 3. Results

### 3.1. Response Rate

**Figure 1:**
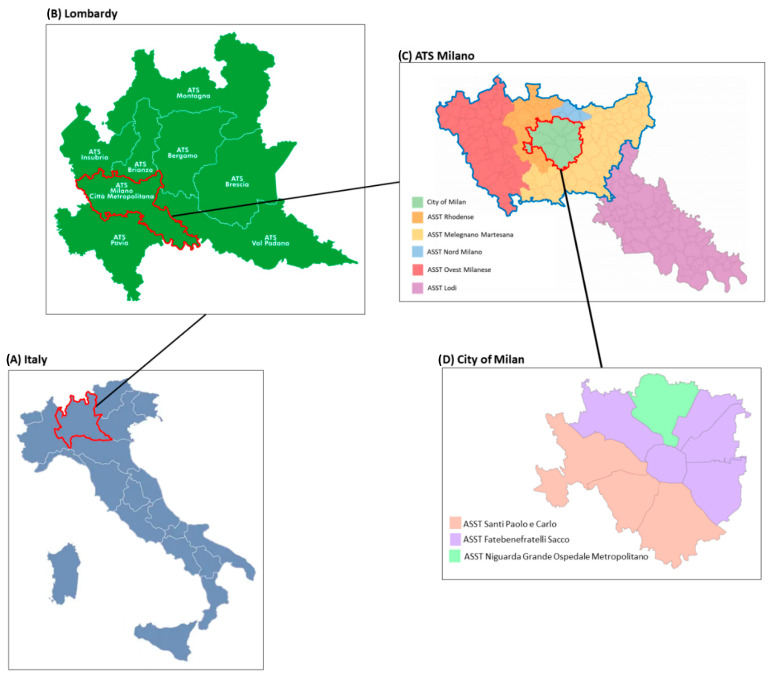
Map of the territory of interest. (**A**) Map of Italy with the Lombardy region marked in red; (**B**) map of Lombardy region with “Agenzia Tutela della Salute” (ATS—Health Protection Agency) Milano marked in red; (**C**) map of ATS Milano with “Aziende Socio Sanitarie Territoriali” (ASST—Territorial Social-Health Authorities) of interest marked in blue and the city of Milan territory marked in red; (**D**) map of City of Milan divided in its three ASSTs.

In the territory of the Metropolitan City of Milan, seven ASSTs of interest were identified (Figure 1): ASST Fatebenefratelli Sacco, ASST Santi Paolo e Carlo, ASST Niguarda Grande Ospedale Metropolitano, ASST Rhodense, ASST Melegnano Martesana, ASST Nord Milano, ASST Ovest Milanese.

Aggregated findings obtained based on the survey responses are reported in Table 1.

All the DAPSS Directors of the seven ASSTs received the survey (delivery rate = 100%). Overall, we had a response rate of 86% (six out of seven participants).

### 3.2. Survey Findings

The participants reported that in the territory of the Metropolitan City of Milan, 37 community houses (with a median of 5 per ASST) were opened. Currently, 13 of them (35%) offer a physiotherapy service (with a median of 2 per ASST). The presence of other rehabilitation professionals (i.e., speech and language therapists and occupational therapists) was recorded in fewer cases (a median of 1 per ASST).

The majority of respondents (five out of six) reported a full-time presence of physiotherapists in the facilities.

The rehabilitation needs of the patients accessing the community houses are reported to be detected mainly by specialists in PRM (five out of six), followed by general practitioners and nurses (three out of six). Only in two facilities, physiotherapists are involved in the evaluation of the rehabilitation need. However, half of respondents reported that physiotherapists are involved in the subsequent multiprofessional assessment. Once the rehabilitation needs are detected, the specialist in the PRM (five cases) or other medical specialists (pneumologists, neurologists, and orthopedic surgeons) redact the individual rehabilitation project more frequently than general practitioners do.

Five out of six respondents state that physiotherapy is a main reason of access to community houses. General practitioners, PRM specialists, rehabilitation units, pediatricians, and pharmacies were the main referrers. Patients accessed the physiotherapy services also through direct access, protected discharges, and ad hoc pathways.

In all the interviewed ASSTs, the physiotherapists operating in the community houses are directly employed by public agencies and are not provided by third-party entities. They are involved in care processes in the field of orthopedic (in five ASSTs), neuromotor (in four ASSTs), respiratory (in three ASSTs), and cardiac (in one ASST) rehabilitation, as well as prevention/health promotion (in two ASSTs) and assistive devices assessment (in two ASSTs).

All the Directors plan to activate more physiotherapy services in the facilities of their competence. Moreover, they express their willingness and availability to collaborate with other ASSTs and stakeholders to set up new physiotherapy initiatives, particularly in prevention and health promotion (four out of six responders).

## 4. Discussion

The current study demonstrated that the Italian Metropolitan City of Milan’s community-based healthcare facilities are increasing, while the number of rehabilitation services is growing slower. Currently, physiotherapists are employed to take charge of patients referred to the community houses in many fields from musculoskeletal to neurological and cardiorespiratory conditions. This is in line with epidemiological data collected by the regional authorities that identified different conditions, such as hypertension, cardiac pathologies, asthma, chronic obstructive pulmonary disease, dementia, Parkinson’s disease, and multiple sclerosis, as the most prevalent in the Metropolitan City of Milan [15]. Moreover, the same conditions could also benefit from preventive interventions, and findings from our survey confirm that physiotherapists could be effectively involved in health promotion and prevention programs dedicated to people experiencing non-acute health conditions (e.g., chronic respiratory disease, cardiovascular prevention, conservative interventions for musculoskeletal disorders) [16,17,18]. Such types of preventive interventions dedicated to chronic health conditions would be mainly aimed at providing effective management outside of the hospital environment, before the pathology arises or worsens [19,20].

Citizens are referred to the services through many pathways that can facilitate the possibility of access. However, PRM specialists and general practitioners are reported to be the referrer in half or fewer of the ASSTs. A stronger integration between these figures and the community facilities could further improve the territorial accessibility to physiotherapy.

In addition, physiotherapists are totally dedicated to taking charge of the patients with scarce involvement in multi-professional needs assessment. The creation of real multi-professional assessment models should be strongly encouraged, to provide the patients tailored responses based on their specific needs.

The COVID-19 pandemic showed how health systems cannot rely on hospital facilities alone but need a well-developed territorial network to be able to meet all the needs of the population, characterized also by different comorbidities, even in times of great crisis. Community- and home-care, also supported by digital technologies [21], can deliver effective and cost-saving interventions particularly for those that experience long-term diseases [22], including post-COVID-19 conditions [23,24].

Moreover, aging, chronic disease, and long-term care put increasing pressure on the healthcare systems of high-income countries [25]. International initiatives call for greater efforts to strengthen rehabilitation in health systems and facilitate its implementation in complex settings [26,27], including primary healthcare [28]. Substantial value can be unlocked by organizing care in the most sustainable way from both the perspective of patients (logistically and financially accessible) and funders (avoiding preventable hospitalization, complications, or inappropriate emergency care referrals) [29,30]. To put it with the European Commission Expert Panel on Effective Ways of Investing in Health, such better organization could specifically improve the safety and quality of life of patients (personal value), reduce the burden of care on caregivers (societal value), minimize the access to inappropriate, expensive care (technical value), and free acute hospital or emergency visits to those patients who can benefit most (allocative value) [31].

Community care enhancement, integrated care pathways, financial incentives, personal health budgets, and advanced technology infrastructures (e.g., telemedicine, telerehabilitation, and digitalized informative systems) are some of the main efforts globally tested to make healthcare safe, better, and sustainable. The community houses need to be understood in this context. All patients who can be diagnosed, treated, and monitored without referring them to big hospitals and acute care facilities are eligible for the community houses. Physiotherapy can help patients prevent the worsening of pre-existing chronic conditions, restore function in patients who underwent surgical interventions and/or traumatic events, or maintain autonomy towards the activities of daily living. Promoting community houses could help optimize health outcomes and the efficiency of healthcare spending by avoiding inpatient or out-of-pocket delivery of services.

On the one side, including and extending this service is crucial to reducing the access gaps and inequalities of post-operative care and rehabilitation. On the other side, making this service available puts further financial pressure on the third payer. The question is therefore whether such a strategy is more effective and more efficient than the alternatives. To answer the question, we need first to see such services spread enough to perform cost-effective evaluations. This study aims foremost to fill the former gap and eventually lay the foundation for the latter investigations.

The current study has limitations. First, the survey cross-sectional design did not allow for the collection of data directly from the facilities. However, it provided preliminary and rapid evidence of the current situation. Another issue could be the context considered, which is limited to the Metropolitan City of Milan and its seven ASSTs. If, on one hand, this single area could not provide comprehensive information about the community house status inside the great inter-regional heterogeneity [32,33], on the other hand, it represents a relevant area of the country, including about 5.4% of the national population. Lastly, our survey lacks information regarding the number and types of patients (e.g., elderly/adults/pediatrics, acute/chronic/frail, dependent/independent, etc.) served by the community houses, the frequency and duration of treatments according to the types of patients, and the types of treatments provided by physiotherapists. While the scope of our survey aimed at mapping the presence of physiotherapy services in community houses, given the current presence of physiotherapists and the anticipated increase in demand for physiotherapy, it is now justified to collect this additional information in further explorations. These explorations may be extended to broader contexts at a regional or national level. Finally, it would be important to better map the territorial communication network allowing general practitioners, medical specialists, healthcare structures, and patients to be informed about the presence of physiotherapy services in the community houses.

## 5. Conclusions

A few years after the establishment of community homes, physiotherapy services are increasingly available in the facilities distributed in the Italian Metropolitan City of Milan. However, more efforts are needed to increase the number of facilities delivering rehabilitation interventions, reinforce the network of medical doctors and healthcare professionals that can refer patients to the community houses, ensure effective interventions, particularly in preventive care, and allow for cost-effective evaluations to be performed across the country. Further studies should be performed to identify differences between regions and national peculiarities and better define the number and the characteristics of the patients who can most benefit from this type of care.

## Figures and Tables

**Table 1:** Surveys results.

Questions	Responses
How many community houses are associated with your ASST	5 (5–6) *
How many of these are defined as “hubs”?	4 (2–5) *
How many hubs provide physiotherapy?	2 (1–4) *
How many hubs provide other rehabilitation services?	1 (1–3) *
In addition to the number of hubs, please specify the type (e.g., speech therapy, occupational therapy, etc.)	Speech therapy; occupational therapy
How many community houses are defined as “spoke”?	2 (0–3) *
How many spokes provide physiotherapy?	0 (0–1) *
How many spokes provide other rehabilitation services?	0 (0–0) *
In addition to the number of spokes, please specify the type (e.g., speech therapy, occupational therapy, etc.)”	NA
Is the physiotherapist or are the physiotherapists present?	Full-time: 5/6 Part-time: 1/6
Who identifies the need for physiotherapy?	PRM specialist: 5/6 General practitioner: 3/6 Nurse: 3/6 Physiotherapist: 2/6 Orthopedic surgeon: 2/6 Neurologist: 1/6 Pneumologist: 1/6
Once the need for physiotherapy is identified, how is it initiated?	Completion of Rehabilitation Project by PRM specialist: 5/6 Completion of Rehabilitation Project by other specialist: 4/6 Request from General Practitioner: 2/6
If you answered “Compilation of Rehabilitation Project by another specialist” in the previous question, please specify which specialist.	Specialist from other Rehabilitation Unit: 1/6 Pulmonologist: 1/6 Orthopedic surgeon/neurologist: 1/6
Is it possible for a user to be referred to a community house because they require physiotherapy?	Yes: 5/6 No: 1/6
If you answered yes to the previous question, who is the referee?	Spontaneous access: 5/6 Protected discharge: 2/6 General Practitioner: 3/6 Pharmacy: 1/6 PRM specialist 2/6 Rehabilitation Units: 2/6 Pediatrician: 1/6 Ad hoc pathways 1/6
Is the physiotherapist or are the physiotherapists involved in the multiprofessional needs assessment?	Yes: 3/6 No: 3/6
Do the physiotherapists have direct collaboration with ASST or third parties?”	Yes: 6/6 No: 0/6
In which area(s) do the physiotherapist(s) work?	Orthopedic: 5/6 Neuromotor: 4/6 Prevention and health promotion: 2/6 Respiratory: 3/6 Cardiac: 1/6 Assistive devices assessment: 2/6
The physiotherapist(s) are most involved in:	Take charge: 6/6 Follow up: 0/6 Multiprofessional assessment: 0/6 Other: 0/6
If the physiotherapist(s) are not present, do you think that your ASST will activate this service in the near future?	Yes: 6/6
If you answered yes to the previous question, in what area would you foresee the employment of the physiotherapist?	Orthopedic: 1/6 Neuromotor: 1/6 Prevention and health promotion: 4/6
Would you also appreciate collaborating or consulting with those who have prior experience in implementing the project mentioned in the previous point?	Yes: 5/6 No: 1/6
Would you appreciate being presented with a project involving the implementation of respiratory physiotherapy intervention in the Community House?	Yes: 6/6
Do you think it would be possible, beyond implementation, to send a questionnaire to the stakeholders of the Community Houses to understand if they deem the activation of a physiotherapy service in them essential?	Yes: 6/6

(*) Data are reported as median and interquartile range. Abbreviations: NA = not available; PRM = physical and rehabilitation medicine.

## Data Availability

The data presented in this study are available on request from the corresponding author.

## Supplementary Materials

The following supporting information can be downloaded at: https://www.mdpi.com/article/10.3390/healthcare12161628/s1, Supplementary Materials S1: STROBE Statement and Checklist for Reporting Results of Internet E-Surveys; Supplementary Materials S2: Original italian version of the survey.
